# Impact of RNA structure on the prediction of donor and acceptor splice sites

**DOI:** 10.1186/1471-2105-7-297

**Published:** 2006-06-13

**Authors:** Sayed-Amir Marashi, Changiz Eslahchi, Hamid Pezeshk, Mehdi Sadeghi

**Affiliations:** 1 Department of Biotechnology, University College of Science, University of Tehran, Tehran, Iran; 2 Faculty of Mathematics, Shahid-Beheshti University, Tehran, Iran; 3 Center of Excellence in Biomathematics, School of Mathematics, Statistics and Computer Sciences, University College of Science, University of Tehran, Tehran, Iran; 4 National Institute for Genetic Engineering and Biotechnology, Tehran-Karaj Highway, Tehran, Iran; 5 Department of Bioinformatics, Institute of Biochemistry and Biophysics, University of Tehran, Tehran, Iran

## Abstract

**Background:**

gene identification in genomic DNA sequences by computational methods has become an important task in bioinformatics and computational gene prediction tools are now essential components of every genome sequencing project. Prediction of splice sites is a key step of all gene structural prediction algorithms.

**Results:**

we sought the role of mRNA secondary structures and their information contents for five vertebrate and plant splice site datasets. We selected 900-nucleotide sequences centered at each (real or decoy) donor and acceptor sites, and predicted their corresponding RNA structures by Vienna software. Then, based on whether the nucleotide is in a stem or not, the conventional four-letter nucleotide alphabet was translated into an eight-letter alphabet. Zero-, first- and second-order Markov models were selected as the signal detection methods. It is shown that applying the eight-letter alphabet compared to the four-letter alphabet considerably increases the accuracy of both donor and acceptor site predictions in case of higher order Markov models.

**Conclusion:**

Our results imply that RNA structure contains important data and future gene prediction programs can take advantage of such information.

## Background

In recent years, complete genomic sequences of many eukaryotic organisms are available and identifying genes in genomic DNA sequences by computational methods has become an important task in bioinformatics. Computational gene prediction tools are now essential components of every genome sequencing project. These programs generally identify potential coding regions by homology searches against databases or by identification of gene structural elements (e.g. start and stop positions and donor and acceptor splice sites) in an unknown DNA sequence. The latter task is routinely done using algorithms trained by observed signals in sequences of known structure.

Ab initio gene prediction methods are based on searching for splice site signals in genomic sequences. The 5' boundary or donor sites of introns in eukaryotes almost always contain the dinucleotide GU, while the 3' boundary or acceptor sites contain the dinucleotide AG. However, because of the common occurrences of these conserved dinucleotides, correct detection of splice sites is not possible if the gene finding algorithm is merely based on the GU-AG rule. Unfortunately, signals around these sites (and especially acceptor signals) are not strictly conserved.

Different methods have been developed for splice site detection, including probabilistic models, neural networks and support vector machines, and techniques based on statistical analyses [1,2]. These methods fundamentally seek conserved motifs or features surrounding the splice sites in training datasets containing known real and decoy (non-real) splice sites. They are generally different in the way that they recognize the dependencies between different positions within a signal sequence. Generally, if we take only the splice signal into account for exon/intron boundary determination, we may obtain many incorrectly predicted (false) splice sites.

In eukaryotes, the vast majority of splicing processes are catalyzed by the spliceosome complex, which has been estimated to contain several hundred different proteins in addition to different snRNAs. These factors are responsible for accurate positioning of the spliceosome on the 5' and 3' splice site sequences [3]. Different experimental evidence suggests that RNA secondary structure can affect the splicing process (see [4] and references therein). In addition, it has been suggested that RNA structure prediction can aid the prediction of human acceptor sites [5] and yeast donor and acceptor sites [6].

Patterson and coworkers [5] applied decision trees and support vector machines as standard machine learning approaches, to improve the prediction of acceptor sites with the consideration of structure metrics (e.g. structure free energy and "maximum helix forming probability") calculated from folding of 100-nucleotide windows around each site/non-site. They reported that consideration of such metrics can result in subtle but significant improvements in the prediction of acceptor sites, while the role of these metrics in the prediction of donor sites was reported to be insignificant.

Our group recently applied neural networks to investigate whether addition of RNA secondary structure information can improve yeast donor and acceptor splice site predictions [6]. We predicted pre-mRNA secondary structures for each "gene" (starting from an AUG and ending in a stop codon). We then converted the structure to a string of two alphabet symbols: stem (*S*) and loop (*L*). Then, we combined these symbols and the four-letter alphabet of nucleotides (A, G, C, U) into an eight-letter alphabet. It was found that both donor and acceptor site predictions based on eight-letter alphabet are noticeably more successful compared to the predictions based on the four-letter alphabet. Our results suggest that eight-letter alphabet contains important information (detected by the networks) which is not present in the four-letter alphabet.

In this study, using Markov models of different orders, we investigated whether the addition of RNA structure information positively influences the accuracy of eukaryotic donor and acceptor site predictions. We first predicted the RNA secondary structure for a 900-base window centered on each splice site, and each non-site (decoy site) and translated it into the eight-letter alphabet, similar to our previous work. Zero-, first- and second-order Markov models were applied for the four-letter and eight-letter alphabets. To demonstrate the accuracy and efficiency of the proposed method, a leave one out cross validation test was performed for each dataset. Our results indicate that when a combination of sequence and structure, i.e. the eight-letter alphabet, is applied to predict splice sites by Markov models, the accuracy of prediction considerably improves. Interestingly, this phenomenon is recognizable in a variety of plant and vertebrate datasets. Therefore, we propose that this approach can be extended to other splice site prediction programs.

## Results and discussion

### Why 900-nucleotide sequences were chosen to feed the RNA structure prediction program?

It is practically impossible to predict the structure of full-length pre-mRNAs in higher eukaryotes because of their large sizes and time limitations. Instead, we chose to select a "window" around each site to feed the RNA structure prediction program.

**Figure 1:**
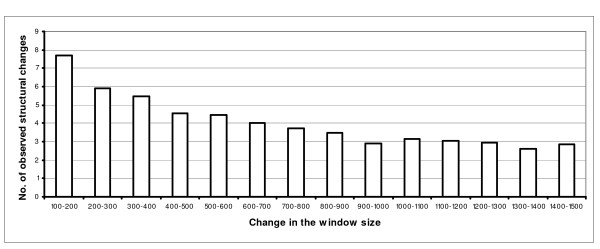
Average number of structural changes in a 21-nucleotide window around 1000 donor GUs. See the text for details.

For 1000 real donor sites in the HsGS dataset (see Methods), we extended the extracted sequences to bigger sizes (i.e. 200, 300, etc centered at each real/decoy donor site) and predicted their structures. Then, we counted the number of *S*→*L *and *L*→*S *changes in a 21-nucleotide window around the donor GU (4 nt in exon + GU + 15 nt in intron). It means that for each of the 1000 donor sites we calculated the number of changes (Hamming distance) between the structure of the 21-nucleotide donor region when folded in a 100-nucleotide window versus a 200-nucleotide window; a 200-nucleotide window versus a 300-nucleotide window, and so on. Figure 1 depicts the average changes vs. the extension of the window. With the enlargement of window from 100-nt to 200-nt, the average number of changes is considerable (in ~36.7% of positions, either of *S*→*L *or *L*↓*S *changes were observed), while after the 900 to 1000 extension few substantial difference found in this value (i.e. it remained about 13.8%) with the extension of the sequence. Roughly the same results were obtained in case of AG sites with the consideration of a 21-nucleotide window around the acceptor site (15 nt in intron + AG + 4 nt in exon).

**Figure 2:**
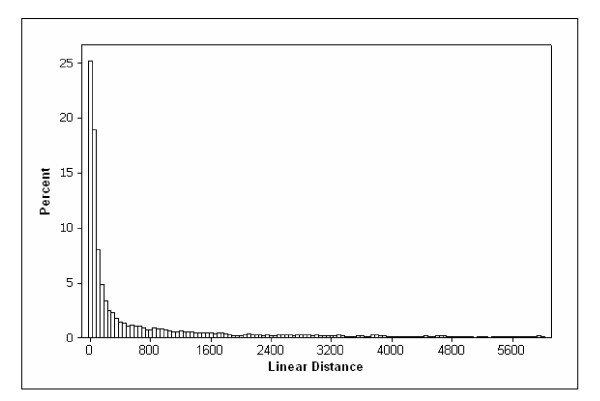
Distribution of predicted linear distances of base-paired nucleotides in RNA sequences. See text for details.

In addition, we predicted the secondary structure of forty 6000-nt sequences (randomly selected from genes in HsGS dataset). The linear distances of all paired bases in the predicted secondary structures were calculated: for nucleotide *i *base-paired to nucleotide *j*, |*i *- *j*| was considered as the linear distance of these two nucleotides. The distributions of these linear distances are shown in Figure 2. A considerable fraction of interactions (~83%) are closer than 900-nt. To capture more possible interactions we had to choose huge windows, for which prediction of structure was extremely slow; for example, ~90% of interactions were closer than 2000-nt. Based on these results, we decided to use 900-nucleotide windows to extract the secondary structures of our splice site datasets from the above-mentioned datasets.

### RNA structure prediction improves splice site prediction accuracy

After prediction of RNA structures of 900-base sequences around each splice site, we translated the normal RNA sequences to eight-letter sequences based on whether or not each nucleotide was in a stem. Then we trained zero-, first- and second-order Markov models with conventional sequence and with eight-letter sequences for prediction of donor and acceptor sites.

Table 1 summarizes the results of this experiment for five different datasets (see Methods). The bold-underlined pairs are those values that show improvements with the application of eight-letter Markov models compared to four-letter models. It is obvious that in case of the second-order Markov model the improvement is exceptionally considerable, while in case of zero-order model this enhancement is not a general phenomenon. It should be noted that Patterson et al [5] reported improvements only in case of the 3' sites.

There are different factors that influence the importance of predicted RNA structure for splice signal detection with different orders of Markov models. First, the number of observations for each dataset at each position of splice signal decreases when higher order Markov models are applied. Thus, the randomness of the training datasets may negatively influence the prediction accuracy. In addition, since RNA base-pairing often occurs between continuous runs of nucleotides longer than 2–3 nucleotides, a zero-order Markov model (which assumes no dependencies between positions) cannot perfectly take the structural information into account. In contrast, the prediction accuracies of first-, and more effectively, second-order Markov models enhance notably.

Since the improvements obtained with the application of eight-letter instead of four-letter alphabet is generally small, it is necessary to verify the observed deteriorations and improvements are statistically significant. As explained in the Methods section, we calculated *p*-values for test of differences in these quantities. Very few cases showed insignificant differences, which means that the observed differences are far from being produced by chance.

In our study, AraClean dataset can be considered as an example of small training datasets, which is usually the case when studying novel genomes; BG570 and HMR195 are examples of genes with poorly conserved signals. Also, note that for the two smallest datasets (i.e. AraClean and HMR195), the improvement is more obvious. For the above datasets, the success of application of eight-letter alphabet in splice site prediction implies that the structure around splice sites are preserved enough to be useful in prediction.

### Log likelihood ratios show conservation of structure at splice sites

It is well-known that in living systems, RNA molecules may fold differently from what is predicted by computational methods. With the best possible prediction software, we completely ignore the importance of RNA-binding proteins in the formation of RNA structures. Nevertheless, if the RNA secondary structure is important as a signal, one can expect preserved structural patterns to be present in the signal sequences, particularly when several previous reports insist on the existence of such structural patterns.

**Figure 3:**
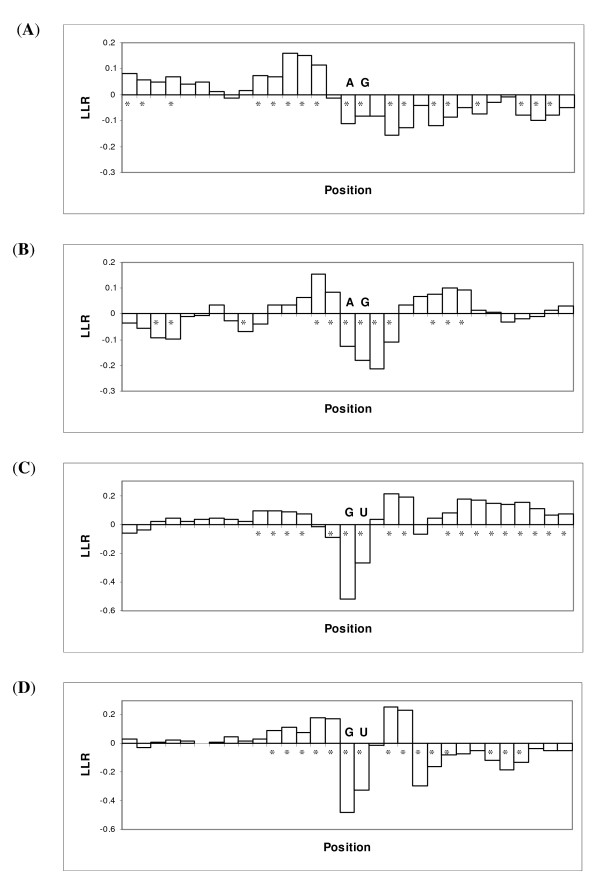
Log likelihood ratio (*LLR *with log-base-2) of formation of loop structure at different positions around splice sites in AtGS and HsGS datasets. The sequences are shown in 5'→3' direction. Asterisked positions are those positions that show a significant difference (*p *< 0.05 based on the test for differences of two binomial proportions) between the frequency of "loops" in real and decoy sites. 3' AtGS (A), 3' HsGS (B), 5' AtGS (C) and 5' HsGS (D).

Figure 3 summarizes the log likelihood ratio (*LLR*, see Methods) of loop formation at different positions of a 31-nt window around splice sites in AtGS and HsGS dataset. *LLR *patterns of AraClean and AtGS, and also *LLR *patterns of BG570, HMR195 and HsGS are qualitatively the same (data not shown). Note that the patterns of *LLR *near the splice sites are roughly the same in human and *A. thaliana*. These patterns are presumably important in the function of spliceosome at different sites, and also confirm the importance of RNA structure in prediction of splice sites. For example, existence of a region upstream of the 3' AG (specially in case of AtGS dataset), which is preferably excluded from being in stem structures, might be related to the fact that existence of stem structures just upstream of the acceptor site inhibits exon ligation [7]. Another important functional sequence is a small region around GU donor sites, which interacts with the U1 snRNA and/or U6 snRNA at the beginning of spliceosome-mRNA interaction. In case of both datasets, it can be seen that the adjacent nucleotides in the left side and also in the right side of the GU dinucleotide prefer to be present in loop structures (see [6]). It seems surprising however, that the GU dinucleotide has a considerable tendency to take part in hybridization with some other parts of the mRNA molecule. We could not establish any functional or sequence similarity between the sites that pair with this GU in the predicted structure. These sites actually were scattered in the 900-nt sequences. Although this can be simply considered as an artifact, it also might be the result of an unknown detection mechanism (e.g. via an RNA-duplex-binding protein), which happens chronologically prior to the binding of U1 snRNA.

One may argue about the impact of RNA structure per se on the splice site prediction. For example, it is previously reported that in case of noncoding RNAs, the distinct statistical properties result mostly from local base-composition bias and not from RNA structure [8].

Table 1 shows that when zero-order Markov model is used, predictions based on eight-letter alphabet generally worsen the splice site identification compared to the four-letter alphabet. In contrast, applying the first-order Markov model fairly improves eight- vs. four-letter splice site predictions, while the use of the second-order Markov model remarkably improves them. If the observed improvements had been simply resulted from the structural differences caused by GC content variations between real and decoy sites, one could have expected to observe the improvements in case of Markov models of any order. Our results imply that the secondary structure information in adjacent nucleotides is in fact the factor that improves the eight-letter-based predictions.

We considered a 21-nt window around each donor or acceptor site, assuming that this window includes all information-containing positions. Figure 3 implies that this cannot be absolutely correct, since far away positions clearly contain species-specific signals. For our datasets, we extended the splice signal window 5 nucleotides from each side. When eight-letter alphabet was applied, predictions of splice sites with this 31-nt window were slightly more successful in comparison with the previous predictions. Altogether, we concluded that the size of the window marginally influences the prediction of splice sites. This is in contrast with four-letter-based predictions, since the information content reduces to zero for distant positions relative to the conserved GU/AG.

### Optimization of RNA structure prediction software for gene prediction: a must?

In this work, we used Vienna software [9] to predict the structure of real and decoy RNA molecules in our datasets. In previous works, Mfold [10] has been exploited to predict RNA structures [5], [6]. The success of these studies suggests that future gene prediction programs may incorporate RNA structure prediction modules. However, the RNA structure prediction programs might not act ideally for gene prediction software, because these programs are designed to find the structure with the least free energy and they test all possible secondary structures; as a result, they are too slow and are not suitable to be associated with gene prediction in genomic sequences, in which a lot of sites should be tested. Therefore, a rapid but approximate structure prediction algorithm might be more useful. Moreover, these programs work best for short sequences and they should be revised to perform better for longer RNA molecules [11]. There are also other algorithms and strategies to predict RNA structure [12]. One should check whether these programs are more useful to be associated with gene prediction programs. Optimized algorithms for RNA structure prediction may significantly improve current gene prediction software.

## Methods

### RNA secondary structure prediction

The Vienna RNA package [9] was used to predict the most stable RNA fold for each sequence. The RNAfold program in the package predicts the minimum free energy structure of a single sequence, based on the algorithm originally developed by Zuker and Stiegler [13]. The predicted structures were converted to a string of two-symbol alphabet (i.e. *S*, *L*) corresponding to whether each nucleotide is paired or unpaired, respectively. Then, with the combination of *L *and *S *symbols and four-letter nucleotide alphabet (i.e. A, U, C, G), each sequence was converted to an eight-letter alphabet sequence. The nucleotide sequences of splice sites (four-letter) and the sequence-structure combination strings (eight-letter) were used for training Markov models (see below).

### Datasets

To evaluate the performance of splice site prediction and comparison of primary sequence vs. prediction based on the combination of sequence and structure, we used some well-known non-redundant datasets with standard donor GUs and acceptor AGs: AraClean dataset with 144 *A. thaliana *genes [14]; AtGS and HsGS datasets with 1323 *A. thaliana *and 1115 *H. sapiens *genes respectively [15]; BG570 dataset with 570 vertebrate genes [16]; and HMR195 dataset with 195 human, mouse and rat genes [17]. In addition, for each donor or acceptor site, 2 GUs and 2 AGs other than the splice site were randomly selected from each dataset (to construct our non-real or "decoy" datasets). In case of each real or decoy site, a 900-base sequence centered at the invariant GU or AG dinucleotides was extracted from the above-mentioned datasets (see below).

### Window size

Patterson et al [5] selected 100-nucleotide sequences centered at each GU or AG dinucleotide, but this might not be the best choice. In a real enormous pre-mRNA molecule in eukaryotes, it is logical to assume that there are many faraway nucleotides base-paired to each other to form secondary structures. We selected 900-nt sequences centered at each real/decoy site; then the RNA secondary structures were predicted for these "windows" (see Results and Discussion).

### Positional Markov models

For devising a positional Markov model, we extracted local 21-nt windows surrounding the candidate splice sites. These local contexts consist of four adjacent nucleotides upstream and fifteen adjacent nucleotides downstream the GU for donor sites. Fifteen adjacent nucleotides upstream and four adjacent nucleotides downstream the AG sites were considered in case of acceptor sites. These segments around donor and acceptor sites are modeled by separate Markov models where the observed state variables are the elements drawn from the four-letter alphabet, or alternatively from the eight-letter alphabet.

We applied zero-, first- and second-order Markov models [18,19]. Briefly, in an *n*th-order Markov model the probability of the observation of a character in the *i*th position depends merely on the characters in its *n *previous positions.

We applied zero, first and second order Markov models. For example, Given a sequence *x *= *x*_1_*x*_2_...*x*_*l*-1_*x*_*l*_, the first order Markov model is such that:

P(x1x2…xl)=P1(x1)∏i=2lPi−1,i(xi|xi−1)     (1)
 MathType@MTEF@5@5@+=feaafiart1ev1aaatCvAUfKttLearuWrP9MDH5MBPbIqV92AaeXatLxBI9gBaebbnrfifHhDYfgasaacH8akY=wiFfYdH8Gipec8Eeeu0xXdbba9frFj0=OqFfea0dXdd9vqai=hGuQ8kuc9pgc9s8qqaq=dirpe0xb9q8qiLsFr0=vr0=vr0dc8meaabaqaciaacaGaaeqabaqabeGadaaakeaacqWGqbaucqGGOaakcqWG4baEdaWgaaWcbaGaeGymaedabeaakiabdIha4naaBaaaleaacqaIYaGmaeqaaOGaeSOjGSKaemiEaG3aaSbaaSqaaiabdYgaSbqabaGccqGGPaqkcqGH9aqpcqWGqbaudaWgaaWcbaGaeGymaedabeaakiabcIcaOiabdIha4naaBaaaleaacqaIXaqmaeqaaOGaeiykaKYaaebCaeaacqWGqbaudaWgaaWcbaGaemyAaKMaeyOeI0IaeGymaeJaeiilaWIaemyAaKgabeaaaeaacqWGPbqAcqGH9aqpcqaIYaGmaeaacqWGSbaBa0Gaey4dIunakiabcIcaOiabdIha4naaBaaaleaacqWGPbqAaeqaaOGaeiiFaWNaemiEaG3aaSbaaSqaaiabdMgaPjabgkHiTiabigdaXaqabaGccqGGPaqkcaWLjaGaaCzcamaabmaabaGaeGymaedacaGLOaGaayzkaaaaaa@5D2B@

in which *P*_1_(x_1_) and *P*_*i*-1*i*_(*x*_*i*_|*x*_*i*-1_) refer to the probability of *x*_1 _at the first position and the conditional probability of *x*_*i *_at position *i *given that *x*_*i*-1 _is at position *i*-1, respectively. In second order Markov model, each symbol depends on the value of the two preceding symbols. The zero order (positional) Markov model is simply a positional weight matrix [19], and probability of each symbol is independent of the other positions. In order to distinguish the false splice sites consisting of the conserved GU and AG dinucleotides, we define a false model *M*^- ^to characterize the signal segments for false splice sites. To use these models for discrimination, we calculated the score as:

S(x)=log⁡P(x|M+)P(x|M−)     (2)
 MathType@MTEF@5@5@+=feaafiart1ev1aaatCvAUfKttLearuWrP9MDH5MBPbIqV92AaeXatLxBI9gBaebbnrfifHhDYfgasaacH8akY=wiFfYdH8Gipec8Eeeu0xXdbba9frFj0=OqFfea0dXdd9vqai=hGuQ8kuc9pgc9s8qqaq=dirpe0xb9q8qiLsFr0=vr0=vr0dc8meaabaqaciaacaGaaeqabaqabeGadaaakeaacqWGtbWucqGGOaakcqWG4baEcqGGPaqkcqGH9aqpcyGGSbaBcqGGVbWBcqGGNbWzdaWcaaqaaiabdcfaqjabcIcaOiabdIha4jabcYha8jabd2eannaaCaaaleqabaGaey4kaScaaOGaeiykaKcabaGaemiuaaLaeiikaGIaemiEaGNaeiiFaWNaemyta00aaWbaaSqabeaacqGHsislaaGccqGGPaqkaaGaaCzcaiaaxMaacqGGOaakcqaIYaGmcqGGPaqkaaa@4A4D@

This model was applied both for the four-letter and the eight-letter alphabet sequences.

Considering the two-letter alphabet of structure (i.e. *L *and *S*), we defined log likelihood ratio (*LLR*) of "Loops" as:

*LLR *= log_2 _(*f*^*r*,*l*^/*f*^*d*,*l*^)     (3)

in which *f*^*r*,*l *^is the frequency of "loops" (i.e. letter *L*) at the *l*th position of real sites and *f*^*d*,*l *^is the frequency at the *l*th position of decoy sites. Positive *LLR *values indicate that the *l*th nucleotide in the window is placed in loops more than what is expected by chance; the reverse is true for negative values.

Since each nucleotide in the real and decoy datasets is folded either as a "loop" or a "stem", performing a "test of two binomial proportions" allows us to see whether the difference between the *L*/(*L+S*) proportions of real and decoy sites is statistically significant. This test was performed using MINITAB^© ^14 software. A *p*-value less than 0.05 was considered significant.

Performance measures

Sensitivity (*Sn*) and specificity (*Sp*) as common measures for determining the accuracy of prediction methods [16] were calculated as:

*Sn *= *TP*/(*TP *+ *FN*)     (4)

*Sp *= *TP*/(*TP *+ *FN*)     (5)

where *TP *is the number of true positives, *FN *is the number of false negatives, *TN *is the number of true negatives and *FP *is the number of false positives. *Sp *is proportion of predicted real sites that are actually real, while *Sn *is the proportion of real sites that have been correctly predicted as real. Since neither *Sp *nor *Sn *alone constitutes good measures of global accuracy, other measures are developed. The preferred measure for global accuracy which has traditionally been used is correlation coefficient, defined as:

CC=(TP×TN)−(FN×FP)(TP+FN)×(TN+FP)×(TP+FP)×(TN+FN)     (6)
 MathType@MTEF@5@5@+=feaafiart1ev1aaatCvAUfKttLearuWrP9MDH5MBPbIqV92AaeXatLxBI9gBaebbnrfifHhDYfgasaacH8akY=wiFfYdH8Gipec8Eeeu0xXdbba9frFj0=OqFfea0dXdd9vqai=hGuQ8kuc9pgc9s8qqaq=dirpe0xb9q8qiLsFr0=vr0=vr0dc8meaabaqaciaacaGaaeqabaqabeGadaaakeaacqWGdbWqcqWGdbWqcqGH9aqpdaWcaaqaaiabcIcaOiabdsfaujabdcfaqjabgEna0kabdsfaujabd6eaojabcMcaPiabgkHiTiabcIcaOiabdAeagjabd6eaojabgEna0kabdAeagjabdcfaqjabcMcaPaqaamaakaaabaGaeiikaGIaemivaqLaemiuaaLaey4kaSIaemOrayKaemOta4KaeiykaKIaey41aqRaeiikaGIaemivaqLaemOta4Kaey4kaSIaemOrayKaemiuaaLaeiykaKIaey41aqRaeiikaGIaemivaqLaemiuaaLaey4kaSIaemOrayKaemiuaaLaeiykaKIaey41aqRaeiikaGIaemivaqLaemOta4Kaey4kaSIaemOrayKaemOta4KaeiykaKcaleqaaaaakiaaxMaacaWLjaGaeiikaGIaeGOnayJaeiykaKcaaa@6881@

### Leave one out cross validation analysis

For each dataset, a leave one out cross validation (LOOCV) analysis was performed, i.e. each site (real or decoy) was removed and the remaining sites were used to train the Markov models and to score (to test) the removed site. In case of each Markov model, using the above scores, a fixed cutoff value was chosen for each dataset in all iterations of the LOOCV tests, so as to maximize the discrimination between real and decoy sites by maximizing *CC*.

In our study, it is important to see whether the application of the eight-letter alphabet results in improvements in the splice signal predictions compared to the conventional four-letter alphabet, and whether the (probable) improvements are statistically significant. To test the significance of the observed differences in the performance measures, for each dataset (real or decoy) we left one sequence out at a time. Then, based on the above mentioned cutoff value, *TP*, *TN*, *FP *and *FN *were determined. Using Equations (4), (5) and (6), the *Sn, Sp *and *CC *values were calculated. With this procedure we obtained distributions for *CC*, *Sn *and *Sp *values for each model. These distributions were then used to investigate whether the observed differences were significant. The level of significance was considered as *p *< 0.001 in the Mann-Whitney test for the difference in the medians of the distributions of four- vs. eight-letter-based performance measures. MINITAB^© ^14 was used to perform the analysis.

## Authors' contributions

All authors participated in the design of the study and interpreting the results. SAM implemented the method. The manuscript was written by MS and SAM. All authors read and approved the final manuscript.

## Figures and Tables

**Table 1:** Comparison of eight-letter vs. four-letter predictions of acceptor and donor sites for different datasets. Where application of eight-letter alphabet enhances the prediction compared to the conventional four-letter alphabet, the data pair is shown in bold.

			**Markov Models:**								
			
			**Zero-order**			**First-order**			**Second-order**		
					
**Dataset**	**Alphabet**	**Site**	***CC***	***Sp***	***Sn***	***CC***	***Sp***	***Sn***	***CC***	***Sp***	***Sn***
**AraClean:**	4-	3'	0.8540*	0.8923	**0.9039**	**0.8821**	**0.8906**	**0.9504**	**0.9011**	**0.9138**	**0.9511**
	8-	3'	0.8536*	0.8911	**0.9048**	**0.9002**	**0.9043**	**0.9611**	**0.9414**	**0.9456**	**0.9744**
											
	4-	5'	0.8832	0.8871	0.9565*	**0.8917**	**0.8989**	**0.9550**	**0.9066**	**0.9019**	**0.9737**
	8-	5'	0.8817	0.8853	0.9565*	**0.9088**	**0.9010**	**0.9781**	**0.9422**	**0.9351**	**0.9874**
											
**AtGS:**	4-	3'	0.7025	**0.8338**	0.8763	**0.7450**	0.8539	**0.8976**	**0.7787**	**0.8769**	**0.9053**
	8-	3'	0.7002	**0.8359**	0.8705	**0.7455**	0.8495	**0.9041**	**0.7943**	**0.8776**	**0.9219**
											
	4-	5'	0.8029	0.8846	**0.9224**	0.8224	**0.8948**	0.9311	**0.8455**	**0.9048**	**0.9440**
	8-	5'	0.7986	0.8787	**0.9252**	0.8222	**0.8997**	0.9250	**0.8576**	**0.9138**	**0.9464**
											
**HsGS:**	4-	3'	0.7848	0.8649	**0.9287**	**0.8167**	**0.8831**	0.9403	**0.8450**	**0.8979**	**0.9524**
	8-	3'	0.7843	0.8635	**0.9299**	**0.8207**	**0.8888**	0.9375	**0.8574**	**0.9085**	**0.9530**
											
	4-	5'	0.8117	**0.8779**	0.9409	0.8449	0.8988	**0.9510**	**0.8740**	**0.9139**	0.9639
	8-	5'	0.8070	**0.8788**	0.9348	0.8446	0.8935	**0.9570**	**0.8842**	**0.9249**	0.9619
											
**BG570:**	4-	3'	0.9002	0.9046	**0.9597**	**0.9163**	0.9276	**0.9570**	**0.9346**	**0.9347**	0.9761
	8-	3'	0.8981	0.8996	**0.9626**	**0.9174**	0.9137	**0.9748**	**0.9427**	**0.9512**	0.9696
											
	4-	5'	0.8845	**0.9056**	0.9369	**0.9105**	**0.9153**	0.9644	**0.9353**	**0.9351**	**0.9780**
	8-	5'	0.8841	**0.9070**	0.9346	**0.9136**	**0.9218**	0.9615	**0.9437**	**0.9406**	**0.9838**
											
**HMR195:**	4-	3'	**0.8812**	**0.8850**	**0.9596**	**0.9054**	**0.9169**	**0.9567**	**0.9406**	**0.9432**	**0.9774**
	8-	3'	**0.8832**	**0.8868**	**0.9603**	**0.9120**	**0.9172**	**0.9661**	**0.9556**	**0.9525**	**0.9885**
											
	4-	5'	**0.8775**	**0.8980**	0.9378	**0.9204**	**0.9290**	0.9645	**0.9532**	**0.9526**	**0.9850**
	8-	5'	**0.8783**	**0.9224**	0.9095	**0.9275**	**0.9458**	0.9558	**0.9633**	**0.9618**	**0.9892**

* Data pairs with insignificant differences (Mann-Whitney test).
